# Incomplete Neutralization and Deviation from Sigmoidal Neutralization Curves for HIV Broadly Neutralizing Monoclonal Antibodies

**DOI:** 10.1371/journal.ppat.1005110

**Published:** 2015-08-12

**Authors:** Laura E. McCoy, Emilia Falkowska, Katie J. Doores, Khoa Le, Devin Sok, Marit J. van Gils, Zelda Euler, Judith A. Burger, Michael S. Seaman, Rogier W. Sanders, Hanneke Schuitemaker, Pascal Poignard, Terri Wrin, Dennis R. Burton

**Affiliations:** 1 Department of Immunology and Microbial Science, International AIDS Vaccine Initiative Neutralizing Antibody Center and Center for HIV/AIDS Vaccine Immunology and Immunogen Discovery, The Scripps Research Institute, La Jolla, California, United States of America; 2 Ragon Institute of Massachusetts General Hospital, Massachusetts Institute of Technology and Harvard University, Cambridge, Massachusetts, United States of America; 3 Department of Infectious Diseases, King’s College London School of Medicine, Guy’s Hospital, London, United Kingdom; 4 Laboratory of Viral Immunopathogenesis, Department of Experimental Immunology, Academic Medical Center, University of Amsterdam, Amsterdam, The Netherlands; 5 Laboratory of Experimental Virology, Department of Medical Microbiology, Center for Infection and Immunity Amsterdam, Academic Medical Center, University of Amsterdam, Amsterdam, The Netherlands; 6 Beth Israel Deaconess Medical Center, Boston, Massachusetts, United States of America; 7 Monogram Biosciences, Inc., South San Francisco, California, United States of America; University of Zurich, SWITZERLAND

## Abstract

The broadly neutralizing HIV monoclonal antibodies (bnMAbs) PG9, PG16, PGT151, and PGT152 have been shown earlier to occasionally display an unusual virus neutralization profile with a non-sigmoidal slope and a plateau at <100% neutralization. In the current study, we were interested in determining the extent of non-sigmoidal slopes and plateaus at <100% for HIV bnMAbs more generally. Using both a 278 panel of pseudoviruses in a CD4 T-cell (U87.CCR5.CXCR4) assay and a panel of 117 viruses in the TZM-bl assay, we found that bnMAbs targeting many neutralizing epitopes of the spike had neutralization profiles for at least one virus that plateaued at <90%. Across both panels the bnMAbs targeting the V2 apex of Env and gp41 were most likely to show neutralization curves that plateaued <100%. Conversely, bnMAbs targeting the high-mannose patch epitopes were less likely to show such behavior. Two CD4 binding site (CD4bs) Abs also showed this behavior relatively infrequently. The phenomenon of incomplete neutralization was also observed in a large peripheral blood mononuclear cells (PBMC)-grown molecular virus clone panel derived from patient viral swarms. In addition, five bnMAbs were compared against an 18-virus panel of molecular clones produced in 293T cells and PBMCs and assayed in TZM-bl cells. Examples of plateaus <90% were seen with both types of virus production with no consistent patterns observed. In conclusion, incomplete neutralization and non-sigmoidal neutralization curves are possible for all HIV bnMAbs against a wide range of viruses produced and assayed in both cell lines and primary cells with implications for the use of antibodies in therapy and as tools for vaccine design.

## Author Summary

Antibodies that potently neutralize a broad range of circulating HIV strains have been described. These antibodies target a variety of sites on the envelope protein of HIV, three copies of which associate to form a trimer that decorate the membrane surface of the virus particle. Some of these antibodies target regions of the envelope protein close to the membrane, some bind to the top of the trimer, others bind via carbohydrates which cover the envelope protein and another subset binds to the same site as the human HIV receptor CD4. Despite effectively blocking 50% of infection at low antibody concentrations, for some particular virus/antibody combinations a proportion of virus particles are resistant to antibody neutralization, even at extremely high concentrations. This phenomenon is called incomplete neutralization and also frequently results in non-sigmoidal dose-response curves when antibody concentration is plotted against the level of virus infection. Previously, antibodies that target the apex of the trimer have been associated with incomplete neutralization and non-sigmoidal curves. In this study we show that representatives from all the groups of antibodies described above result in incomplete neutralization against at least one virus but that the phenomenon is more frequent for those binding the apex and the stalk of the trimer. Resistant populations of virus were seen whether the virus was produced in the natural target of HIV infection (human CD4+ T cells) or engineered human cells more commonly used to produce virus to test antibody function. Understanding this phenomenon is important for the future use of antibodies as therapeutics and for vaccine studies as a resistant population of viruses could result in failure to control the virus infection in patients.

## Introduction

The HIV-1 envelope glycoprotein (Env) spike, the sole target of neutralizing antibodies (nAbs), is a heterotrimer of composition (gp120)_3_(gp41)_3_. The gp120 protein includes about 25 N-linked glycans that comprise almost 50% of its mass [1] and the gp41 protein typically includes four conserved N-linked glycans on the C-terminal half of the ectodomain [2]. While the virus uses glycans as a strategy to escape immune detection, there are several regions of Env that are well established as being vulnerable to broadly neutralizing antibody (bnAb) recognition [3–6]. Three regions are found on gp120: the CD4 binding site (CD4bs), an area of V2 at the apex of the Env spike that includes the glycan at N160 and an area involving V3 that includes glycans forming a high-mannose patch and most particularly a glycan at N332. Recent structural studies show that the V2 apex and high-mannose patch epitopes form a contiguous region at the top of the trimeric Env spike [7,8]. One region is found on gp41 close to the viral membrane and is known as the Membrane Proximal External Region (MPER). In addition, 3 new regions of vulnerability bridging gp120 and gp41 have recently been defined [9–12]. The recognition of each of these neutralizing epitopes by broadly neutralizing monoclonal antibodies (bnMAbs) has variable glycan dependence.

We have previously shown that certain bnMAbs display non-sigmoidal neutralization curves that plateau at <100% for some isolates, [13,14]. In these cases, the behavior has been shown to be partly due to glycan heterogeneity in the Env epitope. Therefore, it is worthwhile to briefly consider each of the bnAb epitopes in turn and, in particular, the role glycans play in each epitope before considering neutralization in more detail. The CD4bs is a conserved region on gp120 involved in receptor binding. The first antibody isolated recognizing this region, b12 [15], remains one of the best studied. However, it is less broad and potent than the more recently isolated CD4bs bnMAbs such as VRC01 [16], PGV04 [17], NIH-45-46, 12A12, 3BNC117 [18] and CH103 [19], which neutralize up to 55–90% of circulating viruses. Considering VRC01 and PGV04, crystal structures of the bnMAbs bound to the gp120 core reveal that both contact the glycan at N276 as part of their epitope but, whereas neutralization of PGV04 is dramatically decreased by its removal, neutralization by VRC01 is enhanced [16,17,20]. This glycan is also required for neutralization by another CD4bs bnMAb, HJ16 [21]. Apart from this glycan, no other glycan has been shown to be so strongly associated with neutralization of virus by CD4bs-targeting bnMAbs, although both VRC01 and PGV04 neutralization is enhanced by the removal of the glycan at N461, and furthermore removal of glycans at N301 or N386 can increase accessibility to the CD4bs [22].

PG9 and PG16 [13,23–25], PGT141-145 [26], CH01-04 [27], PGDM1400 [28] and CAP256-VRC26 [29], target a V2 quaternary site at the apex of Env that includes the glycan at N160. The crystal structure of PG9 bound to a V1/V2 scaffold revealed PG9 makes major contacts with this glycan [25], along with strand C of V1/V2 and the glycan at either N156 (CAP45) or N173 (ZM109). Subsequent studies showed that the PG9 epitope involves a Man_5_GlcNAc_2_ at N160 and high mannose-type or complex-type N-linked glycans at the secondary site (N156 or N173) [30]. Further structural studies with a stabilized Env trimer highlighted that only a single PG9 fragment antigen-binding (Fab) binds to each Env trimer [31]. In addition, molecular modeling and isothermal titration calorimetry studies suggested that PG9 can interact with the N160 glycan on an adjacent gp120 protomer within the antibody-trimer complex [31].

The third region of gp120 targeted by bnMAbs is a set of overlapping epitopes that all appear to involve the glycan at N332 as a significant contributor to antibody binding. Some of the most potent bnMAbs to HIV, PGT121-123, PGT125-7, PGT128, and 10–1074 are amongst those targeting this region as well as 2G12, PGT130-131 and PGT135-137. 2G12 binds the terminal mannose of glycans at N295, N332, N339, and N392. PGT121-123 bind gp120 V1, V3 and several surrounding glycans [7]. The central glycan is the Man_8/9_GlcNAc_2_ glycan at residue N332 but complex or hybrid glycans at N137, N156 and N301 are also involved [7,22]. PGT128 binds glycans at N332, N301 and protein sequence at the C-terminal V3 stem [32]. PGT135 binds glycans at N332, N392, and N386, and protein sequence in V3 and V4 [33].

BnMAbs 4E10, 2F5, Z13e1 and 10E8 recognize the MPER on gp41 [34–36]. While these bnMAbs are not known to make direct contacts with glycans, partial deglycosylation of gp140 has been shown to enhance binding of 4E10 and 2F5 [37]. In contrast, the recently described anti-gp41/gp120 bnMAbs PGT151 and PGT152 have been shown to bind to tri and tetra-antennary complex-type glycans at N611 and N637. These glycan interactions are essential for neutralization as the removal of both completely abrogated neutralization for all isolates tested [9]. The newly identified bnMAb 35O22 has been shown to target a site spanning gp41 and gp120 using glycans N88, N230 and N241 [10]. In addition, a previously described bnMAb 8ANC195 [18] was recently found to bind Env via contacts in gp120 and gp41 within a single protomer [11].

Several bnMAbs have been particularly associated with incomplete neutralization. Notably, the PGT150 series bnMAbs do not always achieve 100% neutralization even when able to potently neutralize the virus under investigation with an IC50 value of less than 1μg/ml. Maximum neutralization ranges from 50–100% with PGT151 and PGT152 achieving less than 80% neutralization against 26% and 20% of 117 viruses tested. Similarly, PG16, and to a lesser degree PG9, have also been shown earlier to display neutralization curves, for some isolates, that plateau at <100% and are non-sigmoidal [13,14]. Incomplete neutralization has also been described for the potent MPER bnMAb 10E8 and this has been associated in part with glycan heterogeneity, particularly at N625 [38].

Incomplete neutralization may have serious consequences for the ability of nAbs to protect against HIV exposure. If transmitted viruses are heterogeneous with respect to neutralization sensitivity, as observed for pseudoviruses and primary viruses grown in PBMCs, then the failure of an antibody to neutralize a population of viruses completely could lead to the establishment of an infection. However, it should be noted that incomplete neutralization has not been reported for serum samples from elite neutralizer donors. In the current study, we were interested in investigating the extent of non-sigmoidal slopes that can plateau at <100% for HIV bnMAbs targeting the four best-studied regions of the Env spike involved in broad neutralization. We found all bnMAbs to be subject to inhibition curves that do not reach 100%, and most bnMAbs also had neutralization curves with non-sigmoidal slopes but bnMAbs targeting certain areas of the trimer were more prone to these effects. Primary isolate and molecular cloned viruses produced in primary cells were also incompletely neutralized, suggesting this phenomenon could be significant *in vivo*.

## Results

### Investigation of incomplete neutralization by bnMAbs against a panel of pseudoviruses

A panel of bnMAbs targeting different regions on Env was tested in a highly quantitative pseudovirus neutralization assay on a panel of 278 viral clones (Fig A in S1 Text). Pseudovirus entry into U87 cells expressing CXCR4 or CCR5 was measured by luciferase activity. Viruses that were neutralized with an IC_50_ >1μg/ml were not further considered because we could not exclude the possibility that at/above this concentration the IC_50_ would be too high for 100% neutralization to be reached by a typical sigmoidal curve at the highest antibody concentration measured (50 μg/ml). For each bnMAb, we determined the maximum percent neutralization (MPN), i.e. the percent at which the neutralization curve plateaus for those viruses neutralized with an IC_50_ <1μg/ml. For the panel of bnMAbs tested, b12, 2G12, PGT136 and PGT137 had a relatively low number of viruses neutralized with an IC_50_ <1μg/ml (Table 1), which may create a small sample bias in the ranking of these two bnMAbs.

**Table 1:** Percentage of viruses neutralized with a maximum neutralization.

bnMAbs	99–100%	96–98%	91–95%	≤90%	total viruses^a^
PGT 122	93	4	3	0	123
PGT 136	93	0	7	0	14
PGV04	92	6	0	2	125
PGT 123	89	3	5	3	122
PGT 121	89	4	5	2	136
PGT 128	88	5	5	2	152
b12	80	16	0	4	25
PGT 127	80	14	4	1	90
PGT 126	76	14	9	2	123
PGT 125	73	19	7	1	104
PGT 130	72	12	6	11	102
PGT 135	62	15	9	15	47
PGT 137	58	26	11	5	19
PG9	54	21	13	11	123
PGT 131	53	19	14	14	73
PGT 142	51	25	14	11	95
PGT 145	51	24	16	9	94
PGT 143	45	26	21	8	85
PGT 141	41	29	23	6	82
2G12	40	44	8	8	25
PG16	35	19	15	31	123
2F5	29	31	24	17	42
PGT 144	20	30	23	27	44
4E10	10	26	27	37	62

^a^ total number of viruses with IC_50_ < 1μg/ml.

### BnMAbs targeting different regions of Env all have some viruses that they neutralize with a plateau significantly <100%, but those that target V2 apex and MPER epitopes do so more often than those that target high-mannose patch

**Fig 1:**
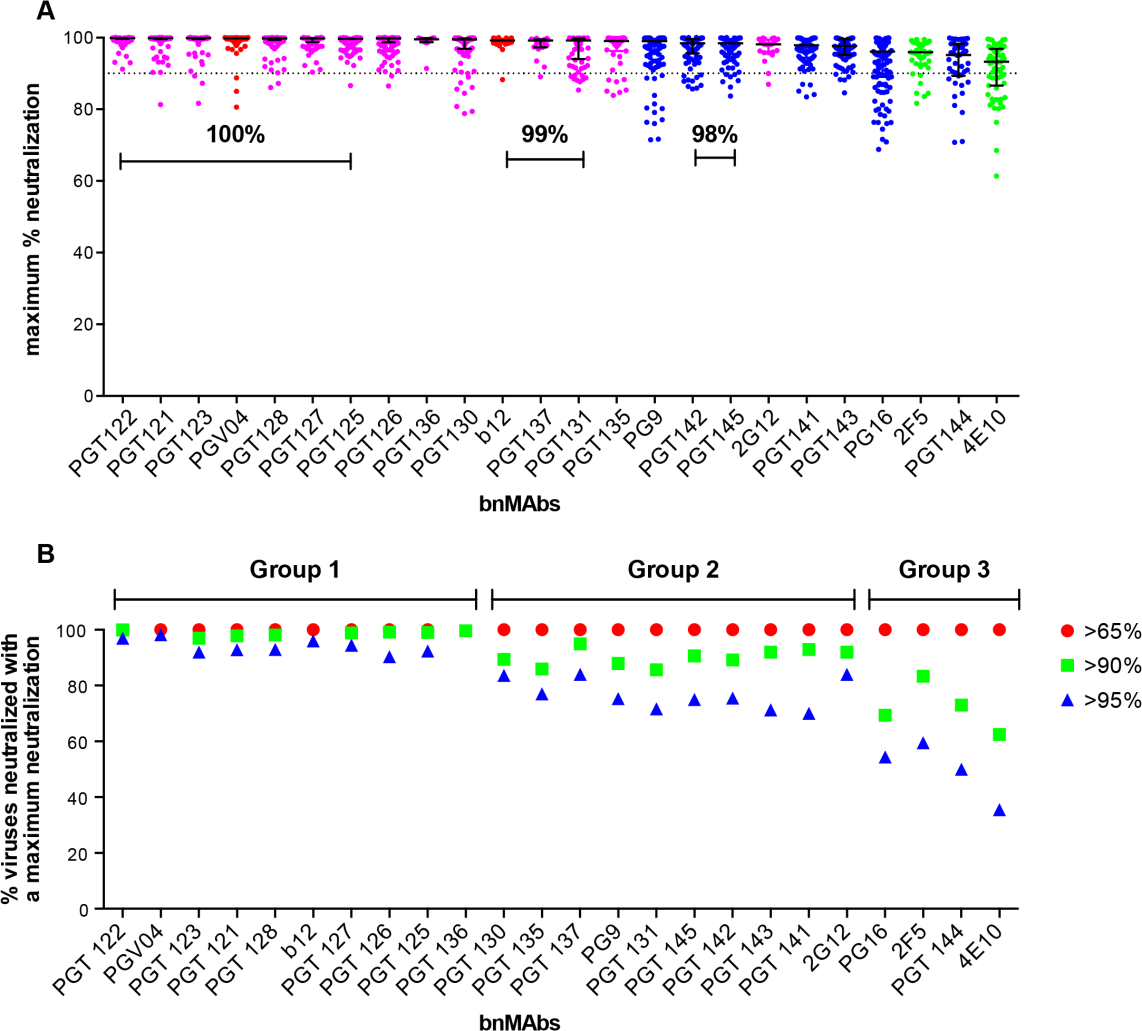
Maximum neutralization activity of bnMAbs in a U87 target cell assay. (A) The bnMAbs are ordered from left to right according to decreasing medians of percent maximum neutralization. Each point on the graph represents the percent at which the neutralization curve plateaued for a single virus. The percentages above the bars group bnMAb medians that have the same values. The dotted line designates 90%. The bars represent the medians and the whiskered bars represent the interquartile range. The data points are colored according to which site the bnMAb targets: high-mannose patch (magenta), CD4bs (red), V2 apex (blue), and MPER (green). (B) Percent viruses neutralized with a maximum neutralization of >95% (blue), >90% (green), and >65% (red). BnMAb groups are indicated above the figure in black and described in the text.

A neutralization curve that plateaus at less than 100% indicates there is a fraction of neutralization resistant viruses that likely express a proportion of Env spikes that are not recognized by the corresponding bnMAb. In Fig 1A, the bnMAbs are ordered from left to right according to decreasing median of the MPN (Fig A in S1 Text). None of the bnMAbs had median MPN values that were <90%, but all of the bnMAbs neutralized at least one individual virus with <90% MPN, except PGT122, PGT127 and PGT136 (Fig 1A). As a group, the bnMAbs that target the high-mannose patch: PGT121-123, PGT125-131, PGT135-137 and 2G12 (Fig 1A, magenta data points) had the highest median MPN values. Of these bnMAbs, PGT121-123 had the highest median MPN, neutralizing viruses to 100% on average, while the other high-mannose patch targeting bnMAbs had median MPN values of 99%. Of the bnMAbs that target the high-mannose patch, 2G12 had the lowest median MPN at 98%. However, 2G12 is an unusual bnMAb because its epitope comprises solely glycans and it is thus more dependent on several glycan sites than the other high-mannose patch bnMAbs [39–41]. PGT130 and 135 also showed less complete neutralization than other high-mannose patch bnMAbs, their median MPN values were equivalent but MPN values as low as 79 and 83% were observed for individual viruses.

The CD4bs bnMAbs also had high median MPN values of 100% and 99% for PGV04 and b12 respectively. These results show that these two CD4bs targeting bnMAbs largely display complete neutralization and, if glycan heterogeneity is the source of incomplete neutralization, this may reflect a relative insensitivity to Env glycan expression, as has been described earlier for bnMAb b12 [14]. However, it should be noted that b12 neutralizes a relatively small proportion of the virus panel, which may have skewed its median MPN value. These data do not allow us to unequivocally conclude that CD4bs bnMAbs as a class are less prone to incomplete neutralization; this would require the evaluation of a larger panel of CD4bs bnMAbs.

Of the bnMAbs targeting the V2 apex (Fig 1A, blue data points), PG9 leads the group with a median MPN value of 99%, although as seen for PGT130 and 135, many individual viruses resulted in MPN values as low as 72%, while median MPN values for PGT 141–145 and PG16 range from 95 to 98%. We have previously reported that, for PG9 and PG16, incomplete neutralization can be attributed, in part, to glycan heterogeneity. JR-FL E168K is an example of a pseudovirus that displays PG9 and PG16 <100% neutralization plateau curves. Notably, PG9 and PG16 neutralization of JR-FL E168K produced in the presence of the glycosidase inhibitor swainsonine reverts the shape of the inhibition curve to a standard curve with a maximum inhibition ∼100% [14].

The gp41 targeting bnMAbs showed the lowest median MPN values (Fig 1A, green data points) at 93% for 4E10 and 96% for 2F5. The inability of 4E10 and 2F5 to reach 100% neutralization for their neutralization curves is, at first glance, inconsistent with the association of glycan heterogeneity with incomplete neutralization since these bnMAbs do not target a glycan as part of their known epitopes. In fact, 2F5 and 4E10 neutralization of the HIV-1 CRF07_BC pseudovirus named FE, increased when single glycans were removed at N197, N301, N355 (gp120) and N625 (gp41) [42]. In addition, deglycosylation of gp140 Env oligomers with PNGase F increased 2F5 and 4E10 binding to the protein [37]. These results could be explained if glycans on gp120 and/or on gp41 affect the accessibility of the MPER in the context of the viral membrane.

Notably, the median MPN value for each bnMAb does not fully describe its propensity for incomplete neutralization. Another way to assess the efficiency of neutralization is to consider the interquartile range of the individual Ab-virus MPN values from which the median arises. Across all bnMAbs tested, regardless of epitope, there was a general trend of increasing interquartile range as the median of MPN decreased (as shown in Fig 1A), in which the interquartile range of each MPN is defined as the scatter of the population and delineates where the middle 50% of the population is located (whiskered bars). PG16, PGT144 and 4E10 showed the largest scatter, indicating these bnMAbs showed the most variability in their capacity to bind heterogeneous envelopes of different viruses.

### BnMAbs can be separated into groups depending on propensity to reach complete neutralization

The percentage of total viruses neutralized at different maximum levels was determined for all viruses that resulted in an IC_50_ <1μg/ml (Fig 1B and Fig B in S1 Text) and the data suggested a natural division of the bnMAbs into three groups. The bnMAbs in Group 1 neutralized >90% of susceptible viruses with MPN values of >95% and include PGT121-123, PGT125-28, PGT136 and PGV04. Group 2 bnMAbs were less effective, resulting in neutralization of between 60–84% of susceptible viruses with MPN values of >95%. Group 2 bnMAbs, include b12, PGT125-127, 130–131, 135,137, 141–143, 145, 2G12 and PG9. The Group 3 bnMAbs, PG16, PGT144, 2F5 and 4E10, were the least able to completely neutralize viruses for which they have an IC_50_ of <1μg/ml. For this group only 36–60% of susceptible viruses were neutralized with MPN values of >95%. The percent of viruses neutralized at >98% was generally relatively similar for somatic variants of a given family although the neutralization activity of these variants against individual viruses can differ substantially[13,26]. PGT128 and PGT145, the broadest and most potent somatic variants of their respective families neutralized the most viruses with MPN values of >98% when compared to their sister clones. In contrast, PGT136, the least broad and potent of the PGT135 family, neutralized the largest proportion of viruses to >98% inhibition when compared to its sister clones. However, due to its lower potency, only a small number of viruses were neutralized by PGT136 with an IC_50_ <1ug/ml so this analysis considered only a restricted subset of viruses, which may explain the relative efficiency of PGT136 at producing complete neutralization.

### BnMAbs targeting V2 apex and the MPER epitopes more often had neutralization curves with slopes that deviated from a sigmoidal slope as compared to bnMAbs targeting the high-mannose patch and the CD4bs epitopes

**Fig 2:**
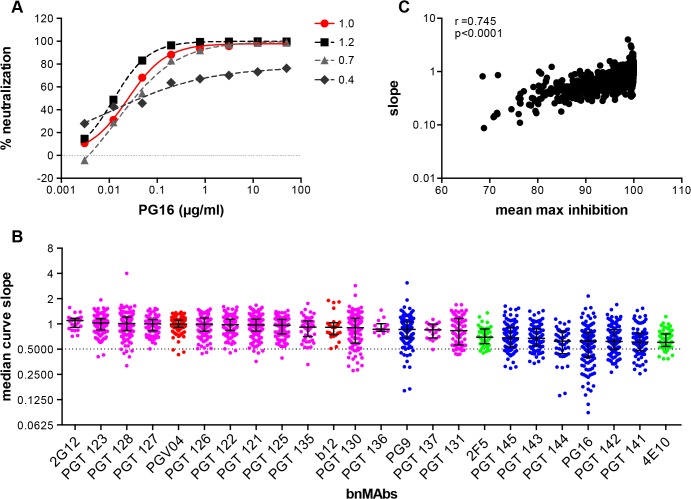
Deviation from standard dose-response curve by bnMAbs in a U87 target cell assay. (A) Representative experiment of PG16 neutralization of HIV pseudoviruses: MGRM-Chronic-B-017.c13, slope = 1.0 (red circle); MGRM-G-022.c02, slope = 1.2 (black square); MGRM-Chronic-B-006.c06, slope = 0.7 (light gray triangle)**;** and MGRM-AG-011.c15, slope = 0.4 (dark gray diamond). (B) The neutralization curve slopes were determined for data from Fig 1A. BnMAbs are ordered according to decreasing medians. The bars represent the medians and the whiskered bars represent the interquartile range. The data points are colored according to which site the bnMAb targets: high-mannose patch (magenta), CD4bs (red), V2 apex (blue), and MPER (green). The dotted line denotes a slope of 0.5. (C) The neutralization curve slope value of each bnMAb for each virus on the y-axis is plotted against the corresponding MPN for each virus-bnMAb pair on the x-axis. Spearman correlation was used for statistical analysis. The Spearman’s rank correlation coefficient was calculated at 0.745 and the P value <0.0001.

While incomplete neutralization suggests the possibility of a mixed population of wild-type envelopes, a percentage of which are not neutralized by a given antibody leading to resistance of a fraction of virions, a non-sigmoidal neutralization curve with a plateau <100% suggests populations of viruses with varying sensitivity to antibody neutralization. A perfect sigmoidal dose-response curve has a slope of 1. The slope of a neutralization curve for a bnMAb that binds all functional Env spikes on a population of wild-type viruses equally well, and neutralizes the corresponding viruses equally well should be close to 1 (Fig 2A and Fig C in S1 Text). A bnMAb that neutralizes a subset of viruses in a wild-type population of viruses less effectively than other viruses in that population may cause a neutralization curve that plateaus at <100% and will have a slope <1 (Fig 2A). A steeper curve with a slope >1 (Fig 2A), suggests either irreversibility of neutralization, cooperativity in binding to trimers or a favorable kinetic neutralization profile which then affects the rate of neutralization of the corresponding viruses. We determined the median curve slopes for the bnMAbs (Fig 2B and Fig D in S1 Text) and found the same general trend was seen for the median slopes as was seen for the median MPN values, and the two had a moderate correlation (Spearman r = 0.745 and a P-value of <0.0001) (Fig 2C and Fig E and F in S1 Text). Again, the high-mannose patch- and CD4bs-targeting bnMAbs conformed most to ideal behavior with median slopes ~1, and V2 apex- and MPER-targeting bnMAbs had median slopes that deviated the most from 1. An exception to this was PG9, which generally had sigmoidal neutralization curves like the high-mannose patch and CD4bs targeting bnMAbs. The median slope of 4E10, 0.6, was the furthest away from a standard slope. The V2 apex-targeting bnMAbs ranged from 0.6 to 0.9. The high-mannose patch bnMAbs ranged from 0.8 to 1.1. None of the bnMAbs had median curve slopes <0.6 and all the bnMAbs had at least one virus for which the slope was <0.72. PGT130 and PGT131 had the largest spread in slope behavior suggesting that neutralization by these bnMAbs was the most sensitive to the isolate context of Env heterogeneity. The median MPN values from each bnMAb-virus pair were also stratified according to viral clade to assess whether certain groups of viral strains are incompletely neutralized by all bnMAbs (Fig J in S1 Text). No clear clade-dependence was observed although some individual viruses are less completely neutralized by all bnMAbs.

### BnMAbs also show comparable incomplete neutralization of some isolates in a TZM-bl cell-based assay

**Fig 3:**
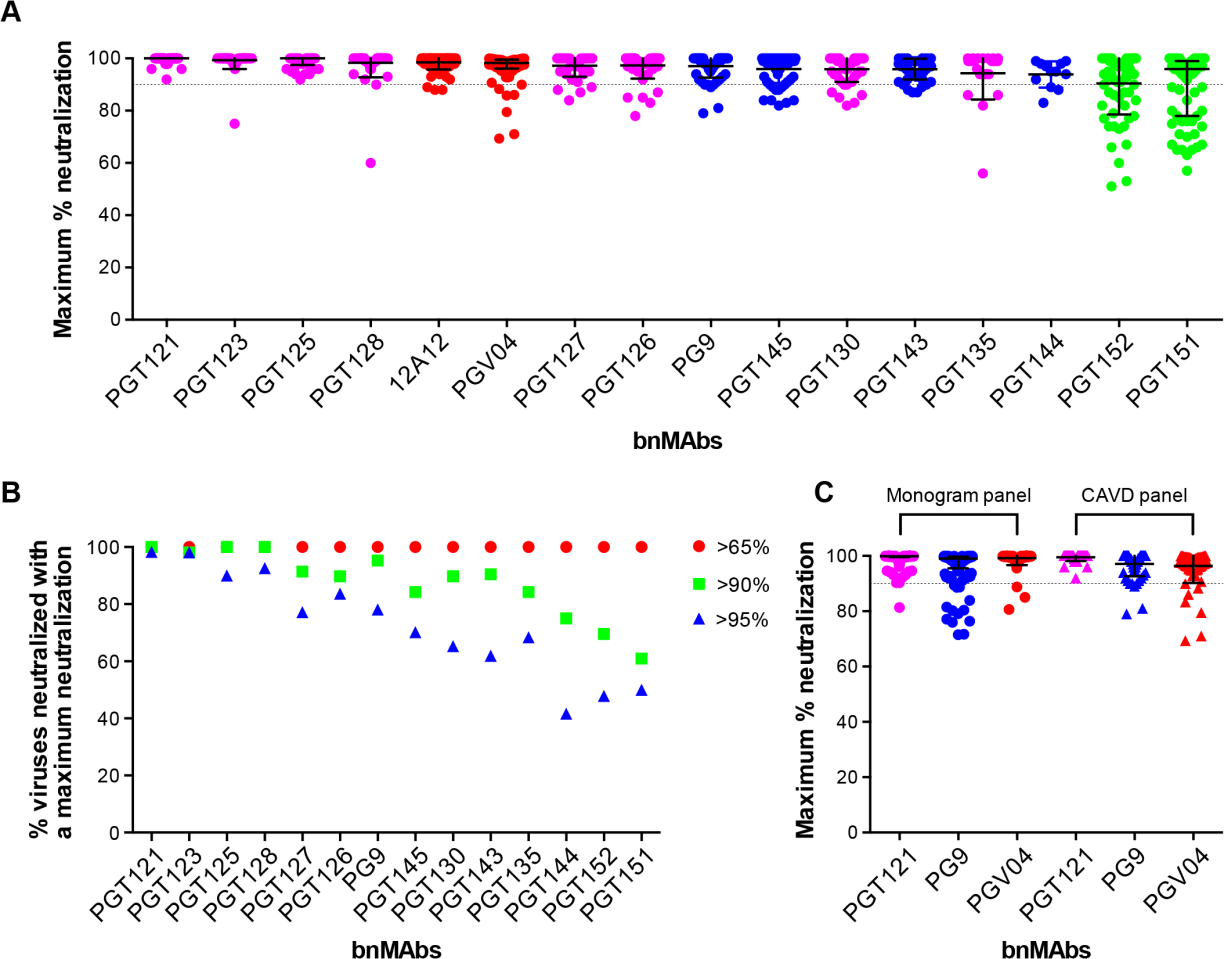
Maximum neutralization activity of bnMAbs in a TZM-bl target cell assay. (A) The bnMAbs are ordered from left to right according to decreasing medians of MPN. Each point on the graph represents the percent at which the neutralization curve plateaued for a single virus. The dotted line designates 90%. The bars represent the medians and the whiskered bars represent the interquartile range. The data points are colored according to which site the bnMAb targets: high-mannose patch (magenta), CD4bs (red), V2 apex (blue), and gp41 (green). (B) Percent viruses neutralized with a MPN of >65% (red), >90% (green), and >95% (blue). (C) Each point on the graph represents the percent at which the neutralization curve plateaued for a single virus against either PGT121 (magenta), PG9 (blue) or PGV04 (red). The assay system used to measure neutralization is indicated above the data points, which are filled circles for the monogram U87 assay and unfilled squares for the CAVD TZM-bl assay.

To investigate a large number of viruses and reliably determine whether complete neutralization had been achieved, the study reported above used a high throughput quantitative pseudovirus assay system and U87 cells expressing CXCR4 or CCR5 as the target cells. However, many more recently described bnMAbs (including the PGT151 family) have been characterized with a TZM-bl cell based assay using a different 117-pseudovirus panel. Therefore, we determined the MPN of bnMAbs using this system. In Fig 3A, the bnMAbs are ordered from left to right according to decreasing median MPN as shown for the U87 cell assay in Fig 1A. The overall level of incomplete neutralization was similar to that seen for the U87 cell assay with median MPN values of the bnMAbs ranging between 94 and 100% and all of the bnMAbs neutralizing at least one virus with an MPN of <90% (Fig G in S1 Text). Furthermore, as previously described for the U87 cell assay, most of the bnMAbs that bind the high-mannose patch resulted in the most complete neutralization across the 117-virus panel with median MPN values close to 100% and the majority of viruses neutralized to >90%. Again, as seen in the U87 assay, when the bnMAbs were divided into groups based on their median MPN of susceptible viruses (Fig 3B and Fig H in S1 Text). PGT121 and 123 were among the bnMAbs with the most efficient neutralization, with most viruses neutralized to between 95–100% (Fig 3B), although some outliers had lower MPN values (Fig 3A). However, individual viruses were neutralized by certain bnMAbs to a much lesser degree (e.g. 56%, 60%, and 75% for PGT135, 128 and 123 respectively) despite an IC_50_ <1μg/ml. Of note, bnMAbs PGT130 and 135 were less able than other high-mannose patch bnAbs to produce high levels of virus neutralization in agreement with the U87 cell assay results shown in Fig 1A. PGT130 resulted in MPN values >90% for many viruses (Fig 3B) but also resulted in a substantial number of less completely neutralized viruses (Fig 3A). PGT135 on the other hand resulted in a disparate pattern of MPN with a very wide range of values (Fig 3A). It should be noted that PGT135 neutralized a lower number of viruses with an IC_50_ <1μg/ml in this 117-virus panel than any of the other bnMAbs except PGT144.

Together with the high-mannose patch bnAbs, the CD4bs antibodies showed a very high tendency to complete neutralization against the 278-virus panel in the U87 cell assay. In line with this, the CD4bs antibody 12A12 resulted in a MPN values of >90% of all but three viruses in the TZM-bl assay (Fig 3A), with a median MPN value of 100%. In contrast, another CD4bs antibody PGV04 resulted in notably less complete neutralization. While the median MPN values for this bnMAb in both assays was close to 100%, more individual viruses were incompletely neutralized in the TZM-bl than in the U87 cell assay (Fig 3A).

The V2 apex bnMAbs were shown in the U87 cell system to have a greater propensity to result in incomplete neutralization than the CD4bs or high-mannose patch binding bnMAbs. Similarly, in the TZM-bl assay PG9 and PGT143-144 resulted in a greater spread of MPN values than PGT121, 123, 125, 127, 128 and 12A12, with many viruses showing between 90–95% neutralization and a number that were neutralized to only between 75–90% (Fig 3B). However, the level of incomplete neutralization by PG9 and PGT143-144 is comparable in this assay to that seen with PGT130, 135 and PGV04 (Fig 3A).

Finally, the two related specificities PGT151 and PGT152, which target a newly described cleavage-dependent gp120/gp41 glycan epitope [9], showed the least complete neutralization with a wide range of MPN values from 57–100% and 51–100% respectively (Fig 3A). Both showed a considerable proportion of viruses with MPN values <90% (Fig 3B). This is consistent with previous findings for these bnMAbs [9].

Although the U87 and TZM-bl studies reported here use different target cells and different virus panels, the trends observed for the bnMAbs tested in both assays are similar (compare Figs 1A and 3A). A direct comparison of results is provided for the bnMAbs PGT121, PG9 and PGV04 in Fig 3C (Fig I in S1 Text). PGT121, which showed among the most complete neutralization in both assays relative to other bnMAbs results in a somewhat greater range of MPN values in the U87-cell/Monogram panel assay than in the TZM-bl/CAVD panel assay. Similarly, PG9, which results in less complete neutralization than PGT121 in both assays, showed a somewhat greater range of values in the U87 cell assay. In contrast, PGV04 results in more complete neutralization in the U87 assay and a smaller range of values as compared to the TZM-bl assay. However, the U87-cell/monogram assay involved 278 viruses and the TZM-bl/CAVD assay used 117. This difference in number of viruses and the divergent viruses used suggest some caution in some of the more detailed results from the two assays.

### BnMAbs also show incomplete neutralization of viruses grown and assayed in human PBMCs

**Fig 4:**
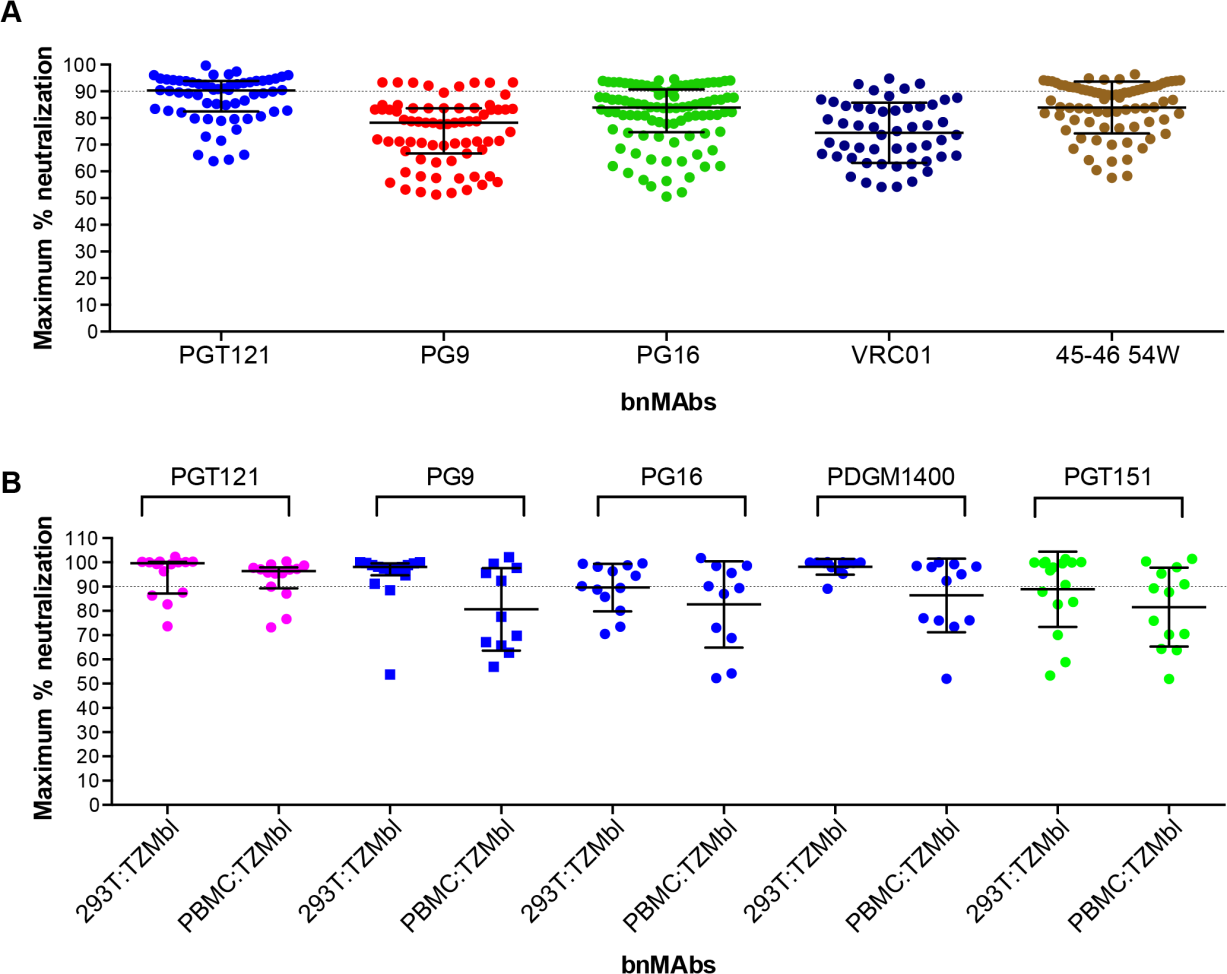
Maximum neutralization activity of bnMAbs in PBMC assays. (A) Each point on the graph represents the percent at which the neutralization curve plateaued for a single PBMC-grown clade B virus assayed in PBMC cells. The dotted line designates 90%. (B) Each point on the graph represents the percent at which the neutralization curve plateaued for a single molecular clone virus against the bnMAbs listed above the data points. Virus clones were produced in both 293T cells and PBMCs as indicated on the x-axis. The data points are colored according to which site the bnMAb targets: high-mannose patch (magenta), CD4bs (red), V2 apex (blue), gp41-gp120 interface (green). The bars represent the medians and the whiskered bars represent the interquartile range.

Previously a subset of bnMAbs targeting the high-mannose patch, V2 apex and the CD4 binding site were characterized for neutralization of molecular clones of virus in a PBMC neutralization assay [43,44]. The clones were isolated from subtype B viral swarms from patients who participated in the Amsterdam Cohort Studies on HIV Infection and AIDS and produced in PBMCs. Here we examine the subset of bnMAbs described above for the ability to neutralize patient-derived virus to 100%. The greater variability inherent in the PBMC neutralization assay renders impossible the definition of MPN with the high degree of precision achievable in the pseudovirus assays and makes reproducibility difficult. Furthermore, for replication competent virus there are multiple rounds of infection during the neutralization assay as compared to a pseudovirus assay where no new viral progeny can be produced. However, with these caveats, we assayed the ability of bnMAbs to neutralize viruses in the PBMC assay (Fig 4A and Fig K in S1 Text). Regardless of epitope, each bnMAb showed a wide variety of MPN values for different viruses. PGT121 produced the least incomplete neutralization with a median MPN of 90% but with values ranging from 64 to 100%. The CD4 binding site antibodies VRC01 and NIH45-46 54W had median MPN values of 74% and 87% respectively. Both PGT121 and NIH45-46 54W, the bnMAbs with the most overall complete neutralization, showed a large group of viruses that were well neutralized (MPN>85%) and a smaller group of viruses forming a “tail” in Fig 4A of dots below 85%. The other bnMAbs neutralized more viruses inefficiently (MPN <85%) and as a result there are more dots in Fig 4A below 85% resulting in a more even distribution of MPN values for these bnMAbs in comparison to PGT121 and NIH45-46W (Fig 4A).

Given our previous work [9,14] describing the influence of glycan heterogeneity on incomplete neutralization by PG9 and PG16, we hypothesized the production of virus in the 293T cell line or primary PBMCs could alter the plateau of a bnMAb neutralization curve against a given virus. One obstacle to investigating this hypothesis is that most of the viruses used in the neutralization panels described in Figs 1–3 are pseudoviruses and therefore cannot be grown in primary cells. Therefore, to study the effects of producer cell type on incomplete neutralization, we used an 18-virus panel of molecular clones, which, unlike the pseudoviruses (Figs 1, 2 and 3) or the patient-derived panels (Fig 4), can be produced either in 293T cells or PBMC cultures and assayed in the TZM-bl neutralization assay. PGT121 was compared to PG9, PG16, PGDM1400 and PGT151 (Fig 4B). The overall trends observed were similar for viruses grown in 293T cells or PBMCs although somewhat greater incomplete neutralization was observed for PBMC-grown viruses. For PGT121, the median MPN was 100% for viruses grown in 293T and 96% for viruses grown in PBMCs; for PG9 the corresponding values were 98% and 88%. Values for the other bnMAbs are summarized in Fig L in S1 Text. As for the large panel of PBMC-grown viruses, overall the median MPN values were lower for PBMC-grown virus than 293T cell-grown virus for the molecular clone panel, although for some individual viruses the opposite was noted i.e. greater MPN values were observed for PBMC-grown molecular clones than the 293T cell-grown virus (Fig M in S1 Text).

## Discussion

We have previously shown that, for a minority of isolates, both PG9 and PG16 have neutralization curves that reach <100% and non-sigmoidal slopes (Walker, 2009) [14]. In the current study, we were interested in determining more on the extent of this phenomenon and therefore we analyzed the neutralization profiles of bnMAbs targeting four major regions of the Env using a large panel of pseudoviruses in a quantitative high-throughput U87 neutralization assay. We found that bnMAbs that target epitopes in the region incorporating the high-mannose patch and two CD4bs bnMAbs neutralized the majority of viruses with MPN values close to 100%. In contrast, bnMAbs targeting the V2 apex region and the MPER more often neutralized viruses with MPN values of <100%. Frequently, neutralization to less than about 95% was associated with non-sigmoidal neutralization curves (Fig 2). The high-mannose patch and CD4bs targeting bnMAbs most frequently showed standard neutralization curves and the V2 apex and MPER targeting bnMAbs showed a higher frequency of non-sigmoidal curves. However, it should be noted that all of the bnMAbs showed less than 95% neutralization and non-sigmoidal neutralization curves for some isolates studied.

To extend these findings, the neutralization profiles of bnMAbs were evaluated with a large panel of pseudoviruses in the TZM-bl assay (Fig 3). Again, the PGT121 and 128 families targeting the high-mannose patch neutralized the majority of viruses with an MPN value close to 100%. The CD4bs bnMAbs 12A12 and PGV04 also neutralized many viruses with MPN values approaching 100%, although PGV04 also resulted in incomplete neutralization with MPN <90% for a sizeable fraction of viruses. Similarly, the V2 apex binding bnMAbs PG9, PGT143-145 more often neutralized viruses with a suboptimal MPN value of <90%. Notably, this study included the addition of two newly described glycan-dependent gp120/gp41 bnMAbs, PGT151-152, which exhibited variable MPN values with a substantial proportion of viruses neutralized to less than 90%. Overall, the results from the two neutralization assays were similar with some indication of a greater tendency to incomplete neutralization in the U87 cell assay than the TZM-bl assay.

The mechanism(s) of incomplete neutralization and non-sigmoidal neutralization curves were beyond the scope of the present investigation but an earlier study on the bnMAbs PG9 and PG16 showed the importance of glycosylation heterogeneity for these features [14], and this heterogeneity correlated with the critical involvement of glycans in the epitopes bound by PG9 and PG16 [7,25,31]. Incomplete neutralization and non-sigmoidal curves are shown here to occur also for other bnMAbs targeting the V2 apex and notably for gp41 bnMAbs. Of these, the PGT151 family depends on tri and tetra-antennary complex-type glycans at N611 and N637 for neutralization function [9], and resulted in the least complete neutralization in the TZM-bl assay, with MPN values as low as 57 and 51% for PGT151 and 152 respectively. In contrast, the MPER Abs have not generally been associated with glycan dependence of binding but this has not been thoroughly investigated [42] and glycan status can influence the accessibility of the MPER to Abs following CD4 engagement, which is when MPER Abs are thought to act [45]. Furthermore, incomplete neutralization by bnMAb 10E8 has been associated in part with glycan heterogeneity [38]. The high MPN values for the bnMAbs targeting the high-mannose patch at first seems inconsistent with their reliance on glycan binding and previous findings showing glycan heterogeneity can result in incomplete neutralization by apex-specific bnMAbs. However, structural studies have shown that PGT128 contacts the N332 glycan on an engineered gp120 outer domain by interacting with the terminal mannose residues of the D1 and D3 arms [32]. This mannose presentation only exists on Man_8/9_GlcNAc_2_ glycans, which suggests that the glycan at N332 may be expressed as relatively homogenous Man_8_GlcNAc_2_ and/or Man_9_GlcNAc_2_ in pseudoviruses [32] and gp120 Env protein [46]. Similarly, PGT122 in complex with a stabilized Env gp140 trimer shows the central N332 glycan is again Man_8_GlcNAc_2._ This homogeneity may be a significant contributor to the ability of bnMAbs that are dependent on the glycan on N332 to bind a high proportion of the envelopes of an isolate effectively and therefore neutralize the vast majority of virions. Furthermore, some bnMAbs recognizing this region can tolerate some heterogeneity in glycan usage [44] and/or use alternate glycan sites [28] that may facilitate tolerance of glycan heterogeneity. Notably, the number of glycan sites on which the high-mannose patch bnMAbs depend for neutralization varies. Consequently those bnMAbs that require more glycan sites such as PGT135/136 had lower median MPN values. The CD4bs bnMAbs are primarily sensitive to a glycan at N276 [20,21] and heterogeneity at this position is a candidate for incomplete neutralization of a virus population. It should be emphasized that there may be mechanisms other than glycan heterogeneity for incomplete neutralization and non-sigmoidal neutralization curves that have yet to be understood; an example would be conformational heterogeneity of the Env trimer [47,48].

While the incomplete neutralization by HIV bnMAbs has herein been clearly demonstrated, it is important to establish how incomplete neutralization in pseudovirus assays relates to anti-viral activity of bnMAbs *in vivo*. As a first step, we investigated the effects on primary viruses *in vitro* by study of the incidence of incomplete neutralization in a large panel of molecular cloned viruses generated from patient swarms from the Amsterdam Cohort Studies on HIV Infection and AIDS. These clones were propagated and assayed in PBMC cultures, which increases the level of variability inherent in the assay and decreases the confidence with which we can define complete neutralization. Furthermore, Abs were titrated from a lower concentration than in the other assay systems which means, although a 1μg/ml cut-off was used as previously described, some Abs may not have reached saturating concentrations in this assay. However, among a large primary virus panel and the five bnMAbs tested there were clear examples of both incomplete neutralization (<90%) and complete neutralization (100%) (Fig 4). Thus, we can conclude that this phenomenon is not an artifact of using 293T cell-produced pseudoviruses for neutralization assays. These data did not directly address whether virus produced in 293T cells or primary cells is more or less susceptible to incomplete neutralization by bnMAbs. To address this we tested a 18-virus panel of molecular clones produced in both cell types and showed that production in PBMCs does not abrogate the incomplete neutralization phenotype (Fig 4 and Fig M in S1 Text) on an individual virus basis. However, the median MPN values for each bnMAb across the 18-virus panel were lower for the PBMC-grown viruses than those for the 293T cell-grown clones. That incomplete neutralization was more frequently observed using PBMC-produced virus on average is a potentially important limitation to the use of 293T cell-grown pseudoviruses for characterizing the likely in vivo efficacy of anti-HIV bnMAbs.

As described, a key question for further study is how incomplete neutralization impacts the ability of bnMAbs to mediate protective effects. Protection has been clearly demonstrated in macaques via passive transfer of many bnMAbs when the challenge virus was completely neutralized [49–55] and as beneficial treatment for pre-existing infection in macaques and humanized mice [56–60]. No systematic studies have been carried out on protection when the challenge virus shows incomplete neutralization by the bnMAb under investigation. However, a recent study found that PG9 provided adequate and typical protection against a challenge virus that showed incomplete neutralization in a TZM-bl assay but essentially complete neutralization in a PBMC assay [61]. Further protection studies will be needed to firmly establish the significance of incomplete neutralization for prophylaxis by bnMAbs and in considering vaccine strategies.

## Materials and Methods

### Ethics statement

Human blood samples from healthy donors were obtained form The Normal Blood Donor service at The Scripps Research Institute. The collection of human blood samples for isolation of PBMCs and subsequent propagation of HIV-1 was approved by the institutional Review Board at The Scripps Research Institute (protocol number HSC-06-4604), all samples were analyzed anonymously. The Amsterdam Cohort Studies on HIV infection and AIDS (ACS) are conducted in accordance with the ethical principles set out in the declaration of Helsinki, and written informed consent was obtained prior to data collection. The study was approved by the Academic Medical Center’s Institutional Medical Ethics Committee.

### Antibodies

2G12, 4E10 and 2F5 (Polymun Scientific, Vienna, Austria) were procured by the IAVI Neutralizing Antibody Consortium. The recombinant bnMAb IgG1 b12 was expressed by the Center for Antibody Development and Production (The Scripps Research Institute, La Jolla, CA) in Chinese hamster ovary (CHO-K1) cells, purified using affinity chromatography (GammaBind G Sepharose, GE Healthcare) and the purity and integrity was checked by SDS-PAGE. PGT 121–123, PGT 125–131, PGT 135–137, PGT 141–145, PGV04, PDGM1400, PGT151-153 and PG9/16, were transiently expressed with the FreeStyle 293 Expression System (Invitrogen). Antibodies were purified using affinity chromatography (Protein A Sepharose Fast Flow, GE Healthcare) and the purity and integrity was checked by SDS–PAGE.

### Neutralization assays

Monogram Biosciences performed neutralization assays using pseudovirus that undergoes a single round of replication as previously described [62]. Briefly, pseudoviruses capable of a single round of infection were produced by co-transfection of HEK293 cells with a subgenomic plasmid, pHIV-1lucu3, that incorporated a firefly luciferase gene and a second plasmid, pCXAS, which expressed an HIV-1 Env clone. Pseudoviruses were harvested 3-days post-transfection and used to infect a U87 cell line expressing co-receptors CCR5 or CXCR4. Pseudovirus neutralization assay using TZM-bl target cells was previously described [63]. For neutralization assays using PBMCs: human PBMCs were obtained from healthy individuals, isolated and stimulated as previously described [44,64]. HIV-1 infectious clone virus stocks were grown and titered on CD8^+^-depleted PBMCS [65]. Virus production was monitored by p24 ELISA (Aalto Bioreagents, Dublin, Eire). For PBMC neutralization assay as previously described [44].

### Statistics

Statistical analyses were done with Prism 5.0c for Mac (GraphPad).

## Supporting Information

S1 Text Fig A, B, C, D, E, F, G, H, I, and L contain tables of data points from which all the main figures were generated. Fig J displays individual MPN values for all bnMAbs and viruses from the 278-virus panel (A) and the 117-virus panel (B) for which the IC50 value <1μg/ml arrayed according to virus strain along the X-axis. Fig M shows MPN values for 18 molecular clone viruses produced in both 293T cells and PBMCs linked by black bars, the bnMAbs tested are indicated above the graph.
(XLSX)
